# Work-Related Psychosocial Factors and Their Effects on Mental Workload Perception and Body Postures

**DOI:** 10.3390/ijerph21070876

**Published:** 2024-07-04

**Authors:** Rodrick Adams, Valentina Nino

**Affiliations:** Department of Industrial and Systems Engineering, Kennesaw State University, Marietta, GA 30060, USA; rodrickpadams@gmail.com

**Keywords:** psychosocial factors, mental workload, body postures, occupational disorders

## Abstract

The perception of work is closely linked to body reactions that facilitate task performance. Previous studies have shown that psychosocial work factors significantly impact employee health on both psychological and physical levels, though their cross-sectional designs limit causal interpretations. In this study, participants performed sitting and standing tasks under four different levels of mental workload. The NASA-Task Load Index (NASA-TLX) assessed mental workload perception across six dimensions, while Rapid Entire Body Assessment (REBA) and Rapid Upper Limb Assessment (RULA) scores evaluated body postures for standing and sitting tasks, respectively. This study examined the effects of alarms, distractions, and time constraints—common psychosocial factors in healthcare environments—on human behavior. We compared NASA-TLX scores with corresponding REBA/RULA scores to evaluate how perceived mental workload affects body postures. One-way ANOVA assessed the impact of experimental conditions on response variables, and Pearson correlation analyses explored the relationships between psychosocial factors and these variables. Results indicated that alarm conditions most negatively impacted mental workload perception and body postures. Temporal demand and effort scores were particularly affected by psychosocial factors in both tasks. Gender influenced physical demand and performance scores (higher in females) for the standing task but did not affect REBA and RULA scores. These findings suggest that organizational and psychosocial factors significantly influence healthcare workers’ behavior, health, and patient safety. Further research is needed to evaluate the specific effects of psychosocial factors on both physical and mental workload to understand the relationship between overall task workload and occupational disorders.

## 1. Introduction

How we move our bodies daily can significantly impact our susceptibility to injuries or disorders of the muscles, joints, tendons, nerves, and spinal discs. When the work environment contributes to the occurrence of those injuries or disorders, they are called work-related musculoskeletal disorders (WMSDs). WMSDs refer to a group of painful disorders caused or exacerbated by work conditions such as recurring forceful tasks, routine lifting of heavy objects, routine overhead work, repetitive motions, and others that are usually sustained in awkward positions [1]. WMSDs arise when the workspace is not designed with the longevity of the human operator in mind. Additionally, WMSDs can occur when an operator performs regular duties with a posture considered “at risk” for developing WMSDs [2]. Some studies have established a link between performing regular work duties and the injury risk associated with the displayed posture [3,4,5,6]. Maulik, De [5] shows a direct correlation between the physical work-related activities of the technicians and the development of WMSDs, with many technicians attributing their musculoskeletal discomfort to their physical work tasks.

Recent studies have shown another link to the development of WMSDs: the mental workload experienced by the operator while performing the work tasks. Research has shown that higher perceived levels of mental workload translate to higher risks of WMSDs due to worse body postures assumed when performing the activities [7]. Mental workload perceptions are influenced by factors that are not overtly inherent to the physical demands of the tasks, such as work environment, job satisfaction, individual characteristics of the employee, and stress levels, among others, that are considered psychosocial factors [8,9]. Currently, there is growing evidence indicating that psychosocial factors in the workplace can significantly impact the occurrence of WMSDs. There is a widespread acknowledgment that factors beyond physical demands, frequently referred to as psychosocial factors, contribute to both the development and persistence of WMSDs [8,9,10,11,12]. For instance, ref. [8] upon finding no correlation between physical demands and the physical symptoms of musculoskeletal issues concluded that the symptoms were instead attributed to psychosocial work stressors and the resulting emotional strain. The model put forth by the authors illustrates that workplace psychosocial stressors can have a substantial effect on employee health, affecting both psychological and physical well-being. 

According to [13] psychosocial factors in the workplace have the potential to trigger psychological stress, which in turn can impact hormonal, circulatory, and respiratory reactions. These reactions could potentially worsen the impact of conventional ergonomic risk factors on employee behavior and mindset, potentially resulting in risky behaviors and subsequently increasing susceptibility to WMSDs [13,14]. Certain studies highlighted not just the significance of balance, but also emphasized the necessity of integrating all components of the system such as workstation design, job demands, mental workload, environmental conditions, and psychosocial factors, along with their interactions when evaluating and (re)designing work systems [15]. suggested that in order to accurately assess workload and gain a comprehensive understanding of the factors influencing human workload, it is essential to break down the overall workload into its constituent components. These components should encompass cognitive, verbal, auditory, visual, and physical workload. This paper explores the extent to which psychosocial factors affect mental workload perception considering six dimensions, as defined by the NASA-Task Load Index (NASA-TLX) [16]: mental demand, physical demand, temporal demand, effort, performance, and frustration, and its effects on the body postures. This study aims to uncover which psychosocial factors influence the perceived mental workload when performing healthcare activities.

The healthcare industry can be considered the pillar of our modern society due to its many advances in increasing the quality of human life. Aside from doctors and specialists who perform the primary operations, the addition of those who assist them, such as nurses and technicians, is a significant piece in keeping the entire operation together and highly functional. Nurses added the benefit of providing frequent care to patients who need it most, drastically decreasing mortality rates and increasing recovery rates [17]. Later, the standard for maintaining top cleanliness was stamped with the addition of a sterile processing department dedicated to preserving sanitation and cleaning efforts; hospitals could improve patient results by moving patients through the chain efficiently by always having sanitized and ready-to-use equipment [18]. This study focuses on the Sterile Processing Department (SPD), which many refer to as “the Heart of the hospital”.

Tasks in healthcare services are highly diverse and require physical, cognitive, social, and emotional abilities. Consequently, task demands in healthcare environments usually exceed human capabilities, resulting in failures to perform procedures safely and increasing the risk of WMSDs [19]. These work-related psychosocial factors influence healthcare workers’ behavior, which affects their health as well as patients’ safety. Studies are needed to evaluate the specific effects of psychosocial factors on physical and mental workload to understand the relationship between tasks’ overall workload and occupational diseases. Previous studies havecorrespondan increased perception of mental workload due to psychosocial factors correspond to worse body postures, and that these perceptions and body postures are also affected for some individual characteristics [7,20]. This study aims to dive deeper and determine which psychosocial factors have the most significant impact on the perception of mental workload and its effect on body postures. Specifically, this research allowed us to measure the impact of alarms, distractions, and time constraints (psychosocial factors common in healthcare environments) on human behavior.

The motivation behind this study is to understand the interconnection of our mind and body and how the perception of our task at hand can impact our physical well-being to assist better those who risk their livelihood in the overall pursuit of providing service to others. We aim to improve the well-being of those assisting in health endeavors to ultimately enhance the well-being of patients who need them. What makes this study so relevant is that in a demanding field such as the healthcare environment, workers frequently complain about body pain, which is most likely attributed to their high-paced work environment, in which they are continually bombarded with multiple alarms, requests, and distractions. By identifying the dimensions of mental workload with the most significant impact on mental workload and postures, our findings may help design interventions to reduce physical risk in high-mental workload environments. The significance of this paper is identifying critical aspects of the workplace environment that have the most substantial effect on the men and women who work there.

## 2. Methods

### 2.1. Participants

Thirty-two volunteers, predominantly young adults aged 25.53 ± 7.96 years, were recruited from a university community comprising 31% staff and 69% students, with a gender distribution of 53% females and 47% males, weight 74.65 (±12.57) Kg, and height 171.5 (±10.20) cm. Recruitment involved disseminating a promotional flyer to students and staff within the university via the university mail list. Participants represented diverse departments and units within the university. Eligibility criteria ensured that participants had not undergone lower or upper extremity surgeries, sustained injuries to these areas within three months prior to the study, or been diagnosed with concussions within a year preceding the study. Participation in the study required a single laboratory session lasting between 1.5 and 2 h. Participants received a monetary incentive upon completion of their participation in the study.

### 2.2. Experimental Design

In the experiment, participants performed two sets of physical tasks (sitting and standing) under four different conditions (baseline, interruptions, timed, and alarms) to simulate the work usually performed in an SPD with the usual psychosocial factors present in a healthcare environment. The standing activity emulated the loading and unloading of surgical trays from and to a rack, an activity usually performed in the decontamination area of an SPD where surgical trays are washed. The sitting task involved assembling a simulated surgical tray, a task typically conducted in the sterilization area of an SPD. Before recruiting participants and conducting experiments, approval was obtained from the University’s Institutional Review Board (IRB). All participants provided informed consent before their involvement.

Psychosocial factors were manipulated through the imposition of a time constraint for task completion, the use of an alarm, and the introduction of interruptions resulting in a total of 8 combinations of treatments. These manipulations were employed to influence and regulate participants’ perceptions of mental workload and examine their impact on the body postures adopted during the experimental tasks. In the baseline condition, participants took an average of two minutes to complete the tasks such as placing containers and lids on a rack or assembling trays. For the time constraint condition, participants were informed they had only two minutes to complete these tasks, creating a sense of urgency despite the consistent time allocation of two minutes to complete the tasks. The auditory condition involved an alarm that began sounding 30 s into the task and continued at 15 s intervals, aiming to enhance the perception of time pressure. This condition also averaged two minutes. In the interruption condition, research personnel interrupted participants by asking for assistance in verifying the assembly of a tray. Participants were instructed to stop their primary task to respond and then return to the primary task, leading to an average completion time of two and a half minutes.

A repeated measure within-subjects experimental design was used so each subject went through all eight treatment conditions. Participants were not directed to use particular lifting techniques or assume specific postures to accomplish the tasks. Physical task demands were kept constant and workstation design was the same for all conditions. Except for the initial completion of the baseline condition, all subsequent conditions were presented to participants randomly to prevent any potential order effects. Following each condition, a rest interval of 2–3 min was allotted to mitigate the accumulation of mental and physical fatigue. During these breaks, participants completed subjective assessments of cognitive workload. More details about the experiment are described in [7].

NASA-Task Load Index (NASA-TLX) was used to assess the perception of mental workload for each condition across six dimensions: mental demand, physical demand, temporal demand, effort, performance, and frustration [16]. The Rapid Entire Body Assessment (REBA) [21] and Rapid Upper Limb Assessment (RULA) [22] were used to objectively evaluate the body postures assumed by the participants while performing the tasks. RULA was used to assess postures while doing the sitting activity, and REBA was used to evaluate postures during the standing activity. TuMeke Ergonomics software [23], a computer-vision joint tracking tool, was used to estimate REBA and RULA scores, with higher scores representing a higher risk of developing musculoskeletal disorders [21,22]. We independently assessed the NASA-TLX dimensions to identify which psychosocial condition significantly impacts mental workload perception. We also juxtaposed NASA-TLX dimensions’ scores with the corresponding REBA or RULA scores to evaluate the effect on body postures.

### 2.3. Analyses

A priori power analysis was conducted using repeated measures ANOVA F-tests, with an effect size *f* = 0.3 (classified as medium according to Cohen’s standards), an error probability α = 0.05, a default correlation among repeated measures of 0.5, four groups, and a total sample size of 30 participants. This analysis yielded a statistical power exceeding 88% for the variables under investigation. Demographic information and general characteristics of the sample such as average age, proportion of males and females, average weight and height were analyzed using descriptive statistics. After verifying the normality assumptions for the response variables REBA, RULA, and NASA-TLX, further analyses were conducted to explore the effects of psychosocial factors on the response variables. Pearson correlation coefficients were calculated to determine the relation between psychosocial factors (baseline, time, alarm, interruption) and NASA-TLX scores of each dimension. After reviewing the descriptive statistics, correlation coefficients, and data plots, analyses of variance (ANOVA) were conducted to evaluate the effects of the psychosocial factors on the perceived mental workload of the participants among the separate NASA-TLX subscales for both activities (sitting and standing). A post hoc test analysis using the Tukey’s method was performed to identify the differences between conditions when the factor effects existed. Minitab Statistical Software version 21.1.0 was used to perform all the statistical analyses in this study. All statistical analyses were evaluated at a significance level of α = 0.05.

## 3. Results

Differences were observed in the effects caused by the psychosocial factors (time, alarms, and interruptions) on the dimensions’ NASA-TLX scores (mental demand, physical demand, temporal demand, effort, performance, and frustration) as well as the postural scores (REBA/RULA). 

The means and standard deviations of the dependent variables are shown in Table 1. Postural analysis scores (REBA/RULA) increased for both activities (standing and sitting) when psychosocial factors were introduced from the baseline, exhibiting the highest scores (worst posture) in the alarm condition, followed by the interruption condition. 

**Figure 1:**
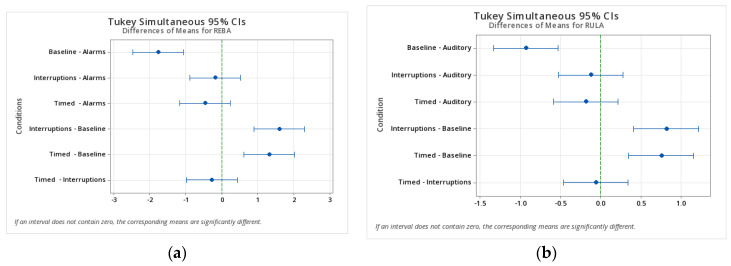
Tukey comparisons between conditions: Standing task (**a**) REBA scores; sitting task (**b**) RULA scores.

We can confirm with 95% confidence (Figure 1) that there was a statistically significant difference in the average postural analysis scores from the baseline condition to the introduction of psychosocial stimuli to the participants. This provides valuable insight into the way we internalize psychosocial factors that, in turn, affect us negatively while working.

Regarding NASA-TLX scores, five out of the six dimensions’ scores increased by at least 20 points while standing and at least 14 points while sitting when compared against the baseline condition, reflecting the higher level of mental workload experienced by the participants in the different psychosocial conditions. We can also confirm with 95% confidence (Table 1 values identified with *) that there was a statistically significant difference in the average NASA-TLX dimensions’ scores, for almost all dimensions in the standing tasks and some in the sitting task, from the baseline condition and the introduction of psychosocial factors such as alarms, interruptions, and time. All increased ratings resulted in higher REBA/RULA scores (worse body postures). Performance dimension scores were the only ones to decrease, reflecting how successful the participants felt they accomplished their tasks, therefore reducing as adverse factors were introduced. The temporal demand dimension, which reflects how hurried the participants felt while performing the tasks, was the highest-rated dimension among the three psychosocial factors, followed by the effort dimension. The lowest scores were a three-way tie between mental demand, physical demand, and frustration for the standing activity, and the physical demand dimension for the sitting activity. 

For the standing task, the highest scores for the physical demand, temporal demand, effort, and frustration dimension were observed in the alarm condition. The mental demand dimension presented the peak scores during the interruption condition, and the performance dimension exhibited the lowest (worst) value for the timed condition. Similar data behavior was observed for the sitting task, with worst scores during the alarm condition for physical demand, temporal demand, effort, and performance dimensions, and peak scores of the remaining dimensions (mental demand and frustration) during the interruption condition. These values indicate that participants experienced the highest levels of mental workload and worst body postures during the alarm and interruption conditions for both activities (standing and sitting). 

With the objective of uncovering which dimension of the mental workload was closely associated with the resulting body posture, a correlation analysis was performed. Specifically, Pearson’s correlation coefficients were estimated to identify any relationship among the NASA-TLX dimensions and the resulting REBA (Table 2) and RULA (Table 3) scores. For the standing task (REBA scores), it was found that each dimension reflected the highest correlation value (moderate to strong correlation) to the resulting posture in the alarm condition, aside from temporal demand which had its highest value in the timed condition. Regarding the impact of psychosocial factors, the alarm condition showed the strongest correlation (0.658) with the mental demand dimension, the interruption condition with temporal demand dimension (0.289), and the timed condition with mental demand dimension (0.425), influencing the resulting postures. 

The sitting activity (RULA scores) showed the alarm condition once again to have the highest correlations (moderate to strong correlation) for mental, physical, and temporal demand dimensions with the resulting posture. Performance presented the highest correlation with RULA scores (0.281) in the interruption condition, effort dimension in the timed condition (0.269), and frustration dimension in the baseline condition (0.164). In terms of the effect of the psychosocial factors (alarms, interruptions, and time) on the resulting posture, all factors presented the highest correlation with the temporal demand dimension (0.509; 0.421; 0.380, respectively). Mental demand and temporal demand dimensions seem to be more closely related with the worst postures assumed when performing the activities. The alarm condition appears to be the one with the highest effect, not only on the perception of mental workload but also on the posture adopted to perform the activity.

Regarding the effect of psychosocial factors on each of the NASA-TLX dimensions, we conducted a one-way ANOVA that revealed a statistically significant effect on all dimensions, except for the performance score, for the standing task, and on temporal demand, effort, and performance dimensions for the sitting task (Table 4). The main effect analysis revealed that temporal demand and effort dimensions (for both activities) are the most negatively (higher scores) affected by the psychosocial factors. We also conducted a one-way ANOVA analysis with the participants’ gender as the main effect. However, out of both activities, standing and sitting, the ANOVA revealed a statistically significant effect only on the physical demand (F = 6.44, *p* = 0.012) and the performance (F = 7.42, *p* = 0.007) dimensions for the standing activity. The main effect analysis also revealed that gender did not have a statistically significant effect on REBA (F = 1.25, *p* = 0.225) or RULA (F = 2.22, *p* = 0.458) scores.

**Figure 2:**
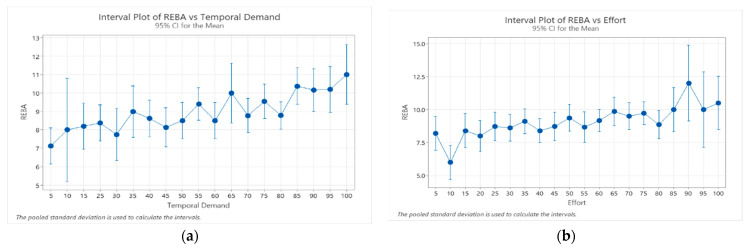
Relationship between REBA scores and temporal demand scores (**a**), and effort scores (**b**).

**Figure 3:**
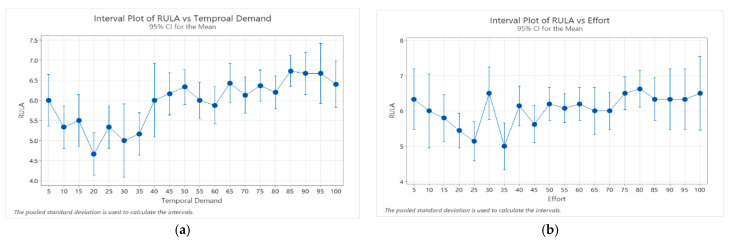
Relationship between RULA scores and temporal demand scores (**a**), and effort scores (**b**).

Since temporal demand and effort dimensions were the most negatively affected by the psychosocial factors, we evaluated the relationship between them and the resulting body postures. It was observed that the highest REBA scores were positive related to highest temporal demand and effort dimension scores (Figure 2). It can be seen that REBA scores increased (worst body postures assumed to perform the standing activity) as the perception of temporal demand and effort also increased. Similar behavior was observed in the sitting activity, where RULA scores increased as temporal demand and effort scores also increased (Figure 3).

## 4. Discussion

This study aimed to delve into the psychosocial factors that most significantly influence the perception of mental workload and its effects on body postures. Essential statistical tools, including scatter plots, correlation coefficients, and analysis of variance (ANOVA) were utilized to analyze the data comprehensively. The alarm condition emerged as the most detrimental to the worker’s experience. In addition to the inherent time pressure associated with task completion, the presence of an alarm seems to significantly exacerbate the perceived mental workload which translates into more awkward and adverse body postures.

The NASA-TLX dimensions most affected by the psychosocial factors in the study (time pressure, interruptions, and alarms) were temporal demand and effort. Both dimensions showed statistically significant differences compared to the baseline condition for both standing and sitting tasks. The temporal demand on average rated higher than all other NASA-TLX dimensions; this remained constant across all four psychosocial conditions. Likewise, temporal demand, effort, and mental demand were the dimensions with the most substantial impact on adverse body postures, as indicated by higher Pearson correlation coefficients for REBA and RULA. These findings reflect how the task pace, the mental load of the task, and the effort required to achieve a desired level of performance influence the body postures adopted when completing activities. Increased perceptions of rush and limited time, higher mental effort required, coupled with the drive to attain high performance, lead to poorer body postures, thereby elevating the risk of developing musculoskeletal disorders. Interestingly, physical demand is not rated highly for poorer body postures, indicating that these adverse postures are not the result of physically demanding tasks but rather of mentally demanding tasks or tasks perceived as mentally demanding. By identifying the dimensions of mental workload with the most meaningful impact on postures, our findings may help design interventions to reduce physical risk in high-workload environments such as the healthcare system.

The results are consistent with previous studies that have found associations between workplace psychosocial factors—such as a hostile work environment, rapid work pace, demanding work schedules, lack of job control, psychosocial stress, monotonous work, social support, work–life balance, job satisfaction—and reported neck, shoulder, and upper body pain [8,10,14]. Reference [24] found associations between psychosocial demands, lack of social support, work condition concerns, and neck–shoulder and low-back complaints in male and female ambulance personnel. Their study included factors like sex, age, smoking, and physical activity. The authors noted that worry, while not pathological, may affect muscle activation and contribute to the development of WMSDs in a cross-sectional context. Oakman et al. [12] investigated the impact of organizational, technical, and individual factors on non-nursing healthcare workers to identify the main predictors of WMSDs risk. They found that age, sex, psychosocial hazards, work–life balance, job satisfaction, and physical demands significantly contributed to levels of discomfort, accounting for approximately 25% of the variance in their model. The aforementioned authors included psychosocial work factors in their studies, underscoring their importance in predicting WMSDs and advocating for a holistic risk assessment approach. However, most did not examine the interaction between psychosocial factors and physical or mental workloads, nor changes in body postures. These cross-sectional studies, conducted under uncontrolled conditions, only identified potential associations without establishing causality and often ignored dual-task conditions, interruptions, distractions, and alarms. Our study conducted dual-task controlled experiments to establish causal relationships using tasks that emulate activities in an SPD. It incorporated healthcare specific distractions such as alarms, interruptions, and time as psychosocial factors to assess their impact on physical and mental workload, evaluating body postures with objective tools.

Gender was a significant factor for NASA-TLX scores in the standing task. When comparing NASA-TLX scores for this activity, females scored higher (43 ± 16) than males (37 ± 17), showing a 18.7% increase in perceived mental workload for females in higher influence in the physical demand and performance dimensions. This might indicate that women experienced higher mental workload than men, especially when psychosocial factors such as noise are present in the work environment, and has been established in previous studies [25,26]. Marchand, Bilodeau [26] affirmed females are more sensitive to noise and therefore experience more noise-induced annoyance and fatigue. It appears that the stress caused by the noise was more pronounced in women than in men. While Grzywacz, Segel-Karpas [25] stated workplace psychosocial exposures are significantly linked to cognitive outcomes, with these effects being particularly pronounced among women. 

The findings indicated that the alarm condition markedly increased mental workload and adverse body postures, a result that underscores the intuitive understanding that any alarm induces a sense of urgency, prompting non-standard bodily movements. Hospitals or healthcare facilities are filled with medical devices that have alarms. Although these alarms are crucial for monitoring patient conditions, they have significant design and usage issues. Previous studies have identified multiple issues associated with clinical alarms and the severe consequences on patients’ outcomes [27,28,29,30]. Based on our findings, we can now add to the list of issues the effect alarms have on the perception of mental workload, adverse body postures, and providers’ well-being. In fact, since the ECRI Institute began publishing its annual “Top 10 Health Technology Hazards” list in 2007, clinical alarms have consistently ranked at or near the top. In 2021, alarms were ranked second [31]. This high ranking persists due to the frequent occurrence of alarm-related incidents and their potentially severe consequences for patients and is based on our findings on healthcare physicians as well. 

The second condition that exhibited the most detrimental effect on mental workload perception was interruptions. Interruptions and multitasking have been identified as significant contributors to clinical inefficiency and errors [32,33,34] and now we can affirm they also affect healthcare practitioner’s mental workload perception and body postures. In a hospital work environment, limiting interdepartmental communication is challenging. However, interventions should be implemented to mitigate the frequency of distractions and disruptions that could be managed at a lower level. One proposed answer is the creation of a liaison position. This role would streamline communication, ensuring that only critical information reaches the healthcare providers, thus reducing unnecessary interruptions, enhancing workflow efficiency, reducing the perception of mental workload, avoiding the assumption of awkward body postures, and therefore enhancing providers’ well-being. 

This study identifies a strong correlation between the perceived difficulty of a task—attributed to the perception of insufficient time, resulting in rushing and increased mental effort to maintain acceptable performance—and the consequent adverse postural effects on the body. The findings underscore the critical relationship between mental workload perception and physical strain, emphasizing the importance of managing perceived mental workload to mitigate its impact on the body due to awkward or harmful postures. This understanding shows us that psychosocial factors play an important role in how we perceive the mental demands required to complete our tasks at hand. These findings may also clarify the links between psychosocial work factors and musculoskeletal disorders and pain [3,7,9,35], as these disorders could result from the body postures adopted during task performance. The observed connection between perceived mental workload and an increased risk of work-related musculoskeletal disorders (WMSDs), evidenced by higher REBA/RULA scores from awkward body postures, underscores the need for interventions targeting healthier organizational design. These interventions should aim to balance the physical demands of tasks with psychosocial work factors, such as alarms and interruptions, particularly within the healthcare system. These findings offer preliminary evidence that factors beyond physical and mental demands can influence task perception, as suggested by previous studies [14,36,37,38,39]. Therefore, it is crucial to consider psychosocial work factors (time to complete the task, level of performance desired or required, task pace) when designing workstations, planning staff schedules, or conducting ergonomic evaluations.

Some interventions could be implemented to mitigate the adverse effects of high mental workload and poor body postures leading to a healthier and more productive workforce. For instance, introduce short, frequent micro-pauses during tasks to encourage posture changes; create liaison roles to streamline communication and reduce unnecessary interruptions; implement systems for regular feedback on workload and ergonomic conditions, allowing staff to voice concerns and suggest improvements; formulate policies that prioritize mental health, including provisions for mental health days, and stress management resources; incorporate technology such as automated systems for routine tasks and ergonomics software that provides real-time feedback on posture and workload; employ virtual reality or simulation training to educate healthcare workers on ergonomic practices and mental workload management in a controlled environment; and utilize wearable devices to monitor physical activity and workload, offering insights into mental workload levels and physical strain that can inform interventions. 

### Limitations and Future Research

Several limitations of this study should be acknowledged. The impact of psychosocial factors may be underrepresented due to the short-term nature of the study. Long-term exposure could yield different interpretations and responses in terms of mental workload perceptions and body behavior. Conducting conditions over a longer period of time might have resulted in higher scores. Future research should focus on integrating longitudinal data to better understand how prolonged exposure to these psychosocial factors influences mental workload perception and body postures, thereby informing more effective and sustainable workplace health interventions and strategies. A central limitation is the reliance on university students, whose perception of mental workload may differ from that of healthcare workers. Future research should extend the investigation to actual workforce scenarios, such as SPD staff, using realistic occupational tasks and equipment. Recent studies have identified issues with REBA and RULA reliability and validity, suggesting that future research should use muscle-specific tasks and biomechanical responses to further understand the connection between body postures and the risk of developing WMSDs. Future research should explore whether other subjective tools, such as SURG-TLX, can more accurately assess mental workload by considering psychosocial factors like distractions, situational stress, and task complexity. The present study did not account for performance variables, which might be influenced by changes in mental workload perception originated by psychosocial factors or the potential relationships with body postures assumed to perform the task. Incorporating performance measures beyond participants’ self-assessments, such as task completion time, error rates, and response accuracy, could provide a more comprehensive understanding of these dynamics. How individuals respond to workplace conditions is a second important group of characteristics that should be considered in future research. For instance, cultural differences in exposure and responses to environmental risks are prevalent. The reactions of Mexican immigrant farmworkers to pesticide risk [40] exemplify how cultural context significantly influences variability in risk adaptation and could impact the implementation of innovative policies. Therefore, individual factors, such as gender, attitudes, anthropometrics, race, cultural differences, and mental health status, should be considered to assess their impact on risk and ergonomic interventions. Future research should also investigate the interrelationship between the factors considered in this study such as psychosocial factors, perceived mental workload, and body postures, and their collective influence on the risk of developing WMSDs. Addressing these gaps is crucial for refining risk profiles across various occupations and recommending thresholds to mitigate risk. Additionally, it would be interesting to evaluate the effects of favorable psychosocial and/or organizational work factors on perceived mental workload and body postures and their consequences that could offer insights into improving workplace safety cultures and reducing occupational disorders. 

## 5. Conclusions

Tasks on healthcare services are highly diverse, requiring physical, cognitive, social, and emotional abilities. Consequently, task demands in healthcare environments often exceed human capabilities, leading to failures in safety performing procedures and increasing the risk within the work environment. These organizational and psychosocial factors significantly influence healthcare workers’ behavior, affecting not only patients’ safety but also workers’ own health and well-being. The significance of this paper lies in identifying critical aspects of the workplace environment that substantially affect healthcare workers. The motivation behind this study is to understand the interconnection between mind and body and how task perception impacts physical well-being. This understanding aims to better support those who dedicate their lives to providing services to others. Our goal is to improve the well-being of healthcare workers, thereby ultimately enhancing the well-being of the patients they serve.

This study measured the impact of alarms, distractions, and time constraints—psychosocial factors common in healthcare environments—on human behavior, specifically examining the body postures assumed during task performance. The findings indicated that alarms have the most detrimental effect on mental workload perception and contribute to adverse body postures, thereby increasing the risk of developing musculoskeletal disorders. Temporal demand and effort were the dimensions most affected by these psychosocial factors, exacerbating the perception of mental workload and resulting in worse body postures.

The study has significant implications for workplace safety, suggesting that psychosocial and organizational factors, along with mental workload management, should be considered to minimize the risk of physical injuries due to poor body postures. In the demanding healthcare field, where workers frequently report body aches, our results indicate that these complaints are likely attributed to their high-paced work environment, where they are continuously bombarded with multiple requests, alarms, and other distractions. Further research is required to investigate the relationship between mental workload and body postures in a more extensive and diverse population. Such research is essential for understanding psychosocial risk factors and assessing the less obvious links between work demands and health outcomes.

## Figures and Tables

**Table 1:** Mean and standard deviations of dependent variables.

Standing					Sitting			
	Mean (SD)				Mean (SD)			
Dimension	Baseline	Timed	Alarms	Interruptions	Baseline	Timed	Alarms	Interruptions
**Mental demand**	16.4 (9.4)	30.5 (18.2) *	36.9 (21.5) *	42.5 (21.2) *	42.3 (23.6)	40.3 (20.8)	43.6 (20.2)	**46.6 (23.3)**
**Physical demand**	19.1 (16.1)	32.3 (21.6)	38.4 (22.6) *	35.3 (23.8) *	17.8 (12.5)	24.5 (19.9)	**29.4 (20.3)**	27.2 (20.7)
**Temporal demand**	30.3 (21.1)	60.6 (23.4) *	69.2 (18.5) *	66.9 (21.8) *	27.8 (17.9)	65.2 (23.0) *	70.2 (21.5) *	65.0 (20.9) *
**Effort**	24.7 (16.3)	52.3 (22.4) *	58.9 (18.8) *	57.9 (18.8) *	33.6 (18.5)	56.3 (25.1) *	61.7 (20.8) *	59.5 (20.5) *
**Performance**	43.6 (23.1)	**42.7 (22.9)**	44.1 (24.4)	45.0 (21.0)	49.2 (29.2)	31.4 (25.7) *	30.9 (25.9) *	40.3 (20.6)
**Frustration**	15.6 (13.06)	31.9 (22.6) *	40.0 (21.7) *	37.5 (22.5) *	28.3 (21.4)	36.3 (23.6)	41.1 (25.0)	**41.7 (25.6)**
**REBA/RULA**	7.8 (1.3)	9.1 (1.5) *	9.5 (1.3) *	9.3 (1.5) *	5.4 (0.8)	6.2 (0.8) *	6.4 (0.7) *	6.2 (0.7) *

**Bold values** indicate highest (worst) scores; * indicates statistically significant compared to the baseline condition (*p* < 0.05).

**Table 2:** Pearson’s correlation coefficient for standing task.

REBA	Mental Demand	Physical Demand	Temporal Demand	Performance	Effort	Frustration
**Baseline**	0.210	0.292	0.252	0.389	0.266	0.393 *
**Alarms**	0.658 *	**0.373**	0.167	**0.474**	**0.362**	**0.438**
**Interruptions**	0.275	0.222	0.289 *	0.120	0.099	0.091
**Timed**	0.425 *	0.277	**0.383**	0.146	0.177	0.159

Highest correlation coefficients for each dimension are the **bold values**; * psychosocial factors.

**Table 3:** Pearson’s correlation coefficient for sitting task.

RULA	Mental Demand	Physical Demand	Temporal Demand	Performance	Effort	Frustration
**Baseline**	0.162	−0.108	0.151	0.015	−0.038	0.164 *
**Alarms**	**0.343**	**0.378**	0.509 *	−0.027	0.091	0.005
**Interruptions**	−0.033	−0.001	0.421 *	**0.281**	0.246	0.044
**Timed**	0.285	0.105	0.380 *	0.153	**0.269**	−0.078

Highest correlation coefficients for each dimension are the **bold values**; * psychosocial factors.

**Table 4:** Statistical significance of psychosocial factors.

	Statistical Analysis (F (*p*-Value))	
	Main Effect of Condition (Psychosocial Factors)	
Dimension	Standing	Sitting
Mental demand	12.15 (0.000) **	0.45 (0.717)
Physical demand	5.15 (0.002) **	2.31 (0.079)
Temporal demand	22.95 (0.000) **	28.18 (0.000) **
Effort	22.05 (0.000) **	11.80 (0.000) **
Performance	0.06 (0.982)	3.60 (0.014) *
Frustration	9.27 (0.000) **	2.15 (0.098)
REBA/RULA	9.99 (0.000) **	10.70 (0.000) **

* (*p* < 0.05), ** (*p* < 0.01).

## Data Availability

The authors do not have permission to share data.
